# Supplementary material for: More than just availability: Who has access and who administers take-home naloxone in Baltimore, MD
Source: PLoS One. 2019 Nov 7;14(11):e0224686. doi: 10.1371/journal.pone.0224686 (PMC6837378; doi:10.1371/journal.pone.0224686)
Supplement: Attachment — Submitted filename: Response to Reviewers.docx [file pone.0224686.s002.docx]

2213 McElderry Street

Second Floor

Baltimore, Maryland 21205

Dear PLOS ONE Editorial Review Team,

We thank the academic editor and reviewers for their thoughtful review of our paper “*More than just availability: Who has access and who administers take-home naloxone in Baltimore, MD*.” We appreciate the suggestions and comments provided by reviewers and have responded to each point raised below.

EDITOR COMMENTS:
Thank you for submitting this manuscript to PlOS One for consideration. We have now received two reviewer reports - both reviewers are enthusiastic about the manuscript and the relevance of the topic for the special issue. While noting that the manuscript is well-written, they do provide some recommendations for how to strengthen the manuscript even further while acknowledging a few additional limitations (see below). I look forward to receiving a revised manuscript that addresses these requests for minor revisions.
**Thank you for your review of the manuscript. As noted in the sections below, we have incorporated the suggestions of the reviewers.**

REVIEWERS COMMENTS:
Reviewer #1: The authors of this manuscript utilized data from a randomized clinical trial conducted in Baltimore, MD to investigate factors associated with obtaining and utilizing naloxone to reverse opioid overdose. This topic is of critical importance since take-home naloxone prevents fatal opioid overdoses if administered in a timely manner. There is strong evidence indicating that the most effective means of reversing opioid overdose with naloxone is to make it available to people who use drugs and are most likely to witness overdose. The authors clearly define their study aims and propose public health interventions to address their findings. Several fairly minor clarifying questions are listed below to improve this strong manuscript.

1) A stated implication of these findings is the need for more aggressive distribution of THN beyond drug treatment settings. Understanding where people access THN in this community is an important priority, particularly for people not engaged in care, and may give insight into how to expand access. Did the study assess where THN was accessed to add to these findings?

**We agree that understanding where people access take-home naloxone (THN) provides an important addition to this paper. The study assessed where participants received training to use Narcan, among those who stated that they had been trained to use Narcan (n=338). Study data found that participants were primarily trained through community outreach programs (36%), recovery programs (34%), and clinics (27%). The minority of participants reported being trained through detention centers (2%), through friends (1%), or other avenues (1%). These study findings are now incorporated into the results section.**

2) On a related note, I continued to consider the definition of and use of the term THN “access” throughout the manuscript. As the title itself suggests, it’s “More than just access: it is who obtains…” This distinction between lack of access (i.e., due to structural barriers or lack of opportunity to access it) vs. not obtaining THN despite access are two different outcomes and could be differentiated more clearly throughout the manuscript. The factors associated with decreased THN “access” – the fear that a person may become aggressive, fear of police threats, and insufficient overdose training – all reflect factors that would be associated with a lower likelihood of obtaining THN rather than factors that would affect likelihood of access. The authors state the limitations of their assessment of access (i.e., “have you ever been prescribed or received a kit containing Narcan?”) and while it may not be possible to distinguish between access to THN vs. obtaining THN given how it was assessed, I would still suggest re-considering the use of the term access for clarity.

**In this paper we have defined access to THN as having received a kit of THN and/or a prescription for THN. While there is no standard definition of access, this definition is aligned with the WHO and NIDA definitions of access (NIDA, 2018, WHO, 2014). We agree with the reviewers’ comments that there is a difference between obtaining THN versus having a prescription for THN. We have therefore revised the manuscript in several ways. First, we amended the title (“More than just availability: Who has access and who administers take-home naloxone in Baltimore, MD”), which removed the word “obtain” to clarify that we did not only examine naloxone possession. Second, we delineate that structural factors affect one’s ability to access THN and individual-level factors may affect one’s desire to access THN in the discussion. We also added a recommendation that future research focus on PWUO who have a prescription for THN and examine factors that influence obtaining THN. As well as suggest that future research examine how people who use opioids maintain a consistent supply after use/loss.**

**NIDA. (2018, June 8). Medications to Treat Opioid Use Disorder. Retrieved from https://www.drugabuse.gov/publications/research-reports/medications-to-treat-opioid-use-disorder on 2019, September 1**

**World Health Organization (WHO). Substance use: Community management of opioid overdose. 2014.**

3) The study focuses on a population of people who use opioids and have a history of injecting drugs. An additional limitation that should be noted is the specification of having injected drugs and findings possibly not being generalizable to PWUO who do not inject. This could be particularly relevant to those people who use prescription opioids who as the authors note in the introduction are less likely to have someone present during an overdose.

**We now include the limitation that study findings may not be generalizable to PWUO who do not inject drugs.**

Minor:
1) Though I feel like it is implied that the authors are referring to intranasal naloxone throughout the paper, it would be helpful to explicitly state this in the introduction. In the context of the question of participants feeling that they have insufficient training, it would also be helpful for the reader to have a brief explanation of what naloxone training entails.
**We have included a description of available formulations of THN and mentioned that the core component of all programs is to enable the effective administration of THN in the management of an opioid overdose in the introduction. In general, intranasal naloxone appears to be more frequently available but due to price injectable naloxone is still distributed.**

2) Though this may not need to be specifically described as a limitation, as a reader I’m curious why so few network members were recruited by index participants in the clinical trial. Is there any reason to think that those people who responded to outreach and advertisement to participate in the study may not be representative of PWUO in Baltimore City?

**The clinical trial did not require index participants to recruit a network member in hopes of boosting enrollment among those not wanting to enroll a network. The low number of network members may also be due to low remuneration for recruiting network members and the requirement that the network members have certain attributes. Network enrollment criteria included being 18 or older and did one of the following:   1) had sex with the index in the past 6 months; 2) injected drugs in the past 6 months; or 3) used drugs with client in the past 6 months.** **Though it is difficult to predict how networks differed from index participants or whether the PWUO in this study represented Baltimore City PWUO more generally, our recruitment at shelters may under-represent middle class PWUO or those who do not keep a primary residence in the city, but rather go between Baltimore city and county lines, which has become more common practice in the current opioid crisis.**

3) Please provide justification for dichotomizing responses to the three questions related to perceived barriers to assist in the event of an overdose.

**The three questions related to perceived barriers to assist at an overdose included perceptions of: insufficient training, that the THN recipient will become aggressive, and that police will threaten people at an overdose scene. These three questions were collected as categorical variables. Based on the distribution and extensive exploratory analyses, we chose to present the dichotomized results in order to enhance interpretability of study findings as the goal of this study was to inform programmatic design. Bivariate sensitivity analyses suggested that dichotomized and categorical study findings were consistent.**

4) P. 14 – “our results suggest that recent injection drug use and having witnessed an overdose are strongly associated with administering THN” – please clarify this statement, as all individuals included in the analyses predicting administration of THN were required to have witnessed an overdose.

**This statement has been revised to read: “Corroborating the findings of Kenney and colleagues, our results suggest that recent injection drug use is strongly associated with administering THN.”**

Reviewer #2:

1) This is a concise, well written description of access to and use of THN naloxone among over 500 at risk individuals in Baltimore, MD. The authors find important correlates and make appropriate recommendations. It would have been interesting to see if there were any associations of actually witnessing a fatality from an overdose. The associations might have been even stronger.

**We agree with the reviewer that it would be interesting to assess the association between witnessing an overdose fatality with THN access and use. This data however was not collected in this dataset and therefore cannot be included in the present article.**

2) The advanced age of the study population may limit generalizability to younger populations and that should be noted.

**We agree and now include older age of study population as a limitation to generalizability within the discussion section.**

Again, we appreciate the input of the reviewers and feel that the article has been strengthened due to their contributions. Thank you for your consideration.

Sincerely,

Lauren Dayton, MSPH
